# Supplementary material for: Y98 Mutation Leads to the Loss of RsfS Anti-Association Activity in Staphylococcus aureus
Source: Int J Mol Sci. 2022 Sep 18;23(18):10931. doi: 10.3390/ijms231810931 (PMC9503621; doi:10.3390/ijms231810931)
Supplement: Supplementary file 1 [file ijms-23-10931-s001.zip › ijms-1899593-supplementary.pdf]

Supplementary Table S1. Potential hydrogen bonds and hydrophobic interactions (< 5 Å) between uL14 and RsfS. Data were calculated for the high-resolution cryo-EM complexes of RsfS with 50S subunit from E. coli (PDB: 7BL4) and *S. aureus* (PDB: 6SJ6).

<table>
<tr><th colspan="4">Potential H-bonds</th></tr>
<tr><th>RsfS S. aureus / E coli</th><th>uL14 S. aureus</th><th>uL14 E. coli*</th><th>Distance (Å)</th></tr>
<tr><td>Arg 68[NH2]</td><td>Arg 107[O]</td><td></td><td>3.10|</td></tr>
<tr><td>Arg 68[NE]</td><td>Asn 110[OD1]</td><td></td><td>2.94|</td></tr>
<tr><td rowspan="2">Glu 70[OE2]</td><td>Met 112[N]</td><td></td><td>3.48|</td></tr>
<tr><td></td><td>Gln 93 [NE2]</td><td>|2.98</td></tr>
<tr><td>Glu 70[OE1]</td><td>Lys 113[NZ]</td><td></td><td>2.96|</td></tr>
<tr><td>Glu 70[O]</td><td>Lys 113[NZ]</td><td></td><td>3.43|</td></tr>
<tr><td>Asn 73 [ND2]</td><td></td><td>Ser 91 [OG]</td><td>|3.08</td></tr>
<tr><td>Trp 77[O]</td><td></td><td>Lys 114 [NZ]</td><td>|2.44</td></tr>
<tr><td>Asp 81[OD2]</td><td></td><td>Arg 108 [NE]</td><td>|2.86</td></tr>
<tr><td>Ala 83[O] / Gly 83[O]</td><td></td><td>Arg 108 [NH2]</td><td>|2.44</td></tr>
<tr><td rowspan="2">Tyr 98[O]</td><td>Arg 97[NH1]</td><td></td><td>2.82|</td></tr>
<tr><td>Arg 97[NH2]</td><td></td><td>3.35|</td></tr>
<tr><td>Tyr 98[OH]</td><td>Pro 93[O]</td><td>Pro 94 [O]</td><td>3.46|3.31</td></tr>
<tr><td>Asn 99[O] / Glu 99 [O]</td><td>Arg 97[NH2]</td><td>Arg 98 [NH2]</td><td>3.44|2.90</td></tr>
</table>

<table>
<tr><th colspan="4">Hydrophobic interactions (< 5 Å)</th></tr>
<tr><th colspan="2">S. aureus</th><th colspan="2">E coli*</th></tr>
<tr><th>RsfS</th><th>uL14</th><th>RsfS</th><th>uL14</th></tr>
<tr><td></td><td></td><td>Ile 33</td><td>Ile 116</td></tr>
<tr><td>Trp 77</td><td>Pro 93, Leu 117</td><td>Trp 77</td><td>Leu 118</td></tr>
<tr><td>Leu 79</td><td>Met 112</td><td>Val 79</td><td>Met 113</td></tr>
<tr><td>Tyr 98</td><td>Pro 93</td><td>Tyr 98</td><td>Leu 118</td></tr>
<tr><td>Ile 100</td><td>Leu 117</td><td></td><td></td></tr>
<tr><td>Leu 103</td><td>Leu 117</td><td></td><td></td></tr>
</table>

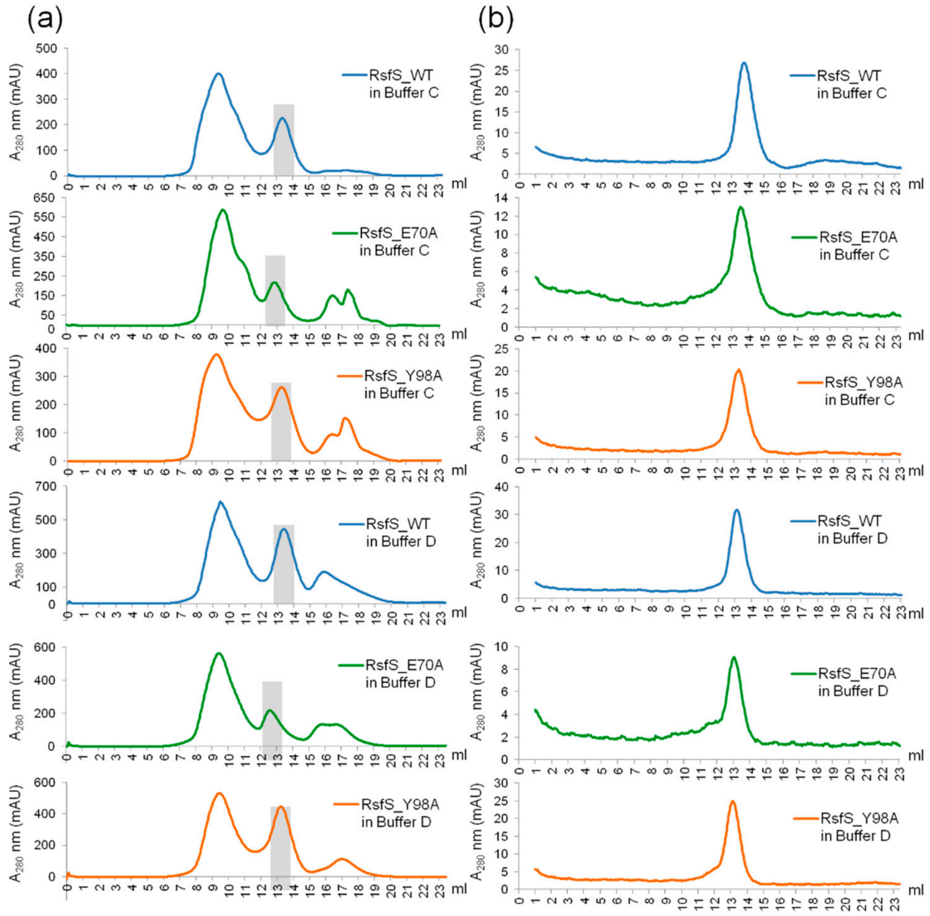

Figure S1. Last step of purification for wild type RsfS (WT) and its mutant proteins (E70A and Y98A). (**a**) Size exclusion chromatography profiles of the proteins in buffers for cell-free experiments (Buffer C) and for centrifugation in the sucrose gradient (Buffer D). The fractions that were collected are shown by grey rectangles. (**b**) Stability and purity control of the collected protein fractions in two different buffers after sample unfreezing.
